# Lab-on-Chip Systems for Cell Sorting: Main Features and Advantages of Inertial Focusing in Spiral Microchannels

**DOI:** 10.3390/mi15091135

**Published:** 2024-09-06

**Authors:** Isabella Petruzzellis, Rebeca Martínez Vázquez, Stefania Caragnano, Caterina Gaudiuso, Roberto Osellame, Antonio Ancona, Annalisa Volpe

**Affiliations:** 1 Physics Department, Università degli Studi di Bari & Politecnico di Bari, Via Orabona 4, 7016 Bari, Italy; isabellapetruzzellis97@gmail.com (I.P.); stefania.caragnano@poliba.it (S.C.); antonio.ancona@uniba.it (A.A.); 2 Institute for Photonics and Nanotechnologies (IFN), National Research Council, Piazza L. da Vinci 32, 20133 Milan, Italy; roberto.osellame@cnr.it; 3 Institute for Photonics and Nanotechnologies (IFN), National Research Council, Via Amendola 173, 70125 Bari, Italy; caterina.gaudiuso@cnr.it

**Keywords:** cell sorting, inertial microfluidics, particle manipulation, Lab-on-Chip

## Abstract

Inertial focusing-based Lab-on-Chip systems represent a promising technology for cell sorting in various applications, thanks to their alignment with the ASSURED criteria recommended by the World Health Organization: Affordable, Sensitive, Specific, User-friendly, Rapid and Robust, Equipment-free, and Delivered. Inertial focusing techniques using spiral microchannels offer a rapid, portable, and easy-to-prototype solution for cell sorting. Various microfluidic devices have been investigated in the literature to understand how hydrodynamic forces influence particle focusing in spiral microchannels. This is crucial for the effective prototyping of devices that allow for high-throughput and efficient filtration of particles of different sizes. However, a clear, comprehensive, and organized overview of current research in this area is lacking. This review aims to fill this gap by offering a thorough summary of the existing literature, thereby guiding future experimentation and facilitating the selection of spiral geometries and materials for cell sorting in microchannels. To this end, we begin with a detailed theoretical introduction to the physical mechanisms underlying particle separation in spiral microfluidic channels. We also dedicate a section to the materials and prototyping techniques most commonly used for spiral microchannels, highlighting and discussing their respective advantages and disadvantages. Subsequently, we provide a critical examination of the key details of inertial focusing across various cross-sections (rectangular, trapezoidal, triangular, hybrid) in spiral devices as reported in the literature.

## 1. Introduction

Since the latter half of the last century, the scientific community has recognized the need to optimize existing chemical, biological, and clinical analysis techniques.

Cell sorting is one of the primary purposes in cell analysis and involves the analysis of cell samples and differentiation into their respective subcellular components within a heterogeneous mixture. The applications of this technique range widely from the study of bacteria and pathogens in the food and environmental sectors [1,2] to the cultivation of stem cells for tissue and organ regeneration [3,4] and the separation of tumor cells from blood sample for early cancer diagnosis [5,6,7,8]. For example, the possibility of isolating stem cells from bone tissue with high purity can improve proliferative and regeneration capacity; also isolating circulating tumor cells (CTCs) from blood cells (BCs) enables cancer diagnosis in the initial stages [9] considering CTC isolation a real-time “liquid biopsy”. However, CTCs are extremely rare in a blood sample, making their isolation and characterization an extreme technological challenge [10,11].

It is important to consider all practical aspects involved when choosing a sorting technique. First, ease of operational execution, low energy requirements for performing the tests, high separation speeds and minimized biological risk for the operator could improve the operational dynamic and reduce the time required. Then, versatility, accuracy, sensitivity, and ability to analyze small amount of biological fluids consisting of a large variety of cells make the technique chosen suitable for different applications and biological matrixes [12]. 

The need to integrate the earlier-mentioned properties into a single separation technique has led researchers to study and design different cells sorting systems; for example, miniaturized devices, based on microfluidic principles, can address this aim. Compared to conventional sorting techniques, like fluorescence-activated cell sorting (FACS) [13], magnetically activated cell sorting (MACS) [14], and centrifugation methods [15], which faced some technical limits, recent microfluidic techniques, applied to cell extraction, offer numerous advantages. Conventional sorting techniques require labeled cells, long processing times, high operating costs and energies and bulky equipment despite offering high efficiency [12]; by contrast, recent microfluidic techniques allow label-free cell sorting, reduced time and power operation, sample volumes and lower equipment fabrication costs thanks to the use of miniaturized devices. Label-free cell sorting techniques rely on cell characteristics such as physicochemical, immunological, and functional properties (size, volume, density, refraction index, membrane potential, pH, electrical impedance, and charge) [16]. 

These techniques can be categorized into (i) active separation and (ii) passive separation. Active separation technologies involve external forces, such as electric or electromagnetic fields, requiring high space, expensive or bulky external generators often offering a low throughput. These include dielectrophoresis (DEP) [17], magnetophoresis (MAP) [18], acoustophoresis [19], and optical tweezers [20]. By contrast, passive separation techniques, do not require external forces; instead, they rely on the microchannel geometry and intrinsic hydrodynamic properties to achieve cell sorting, generally offering higher throughput than active techniques. Cell throughput is defined as the number of cells that can be processed in the time it takes to perform the sorting. In active techniques, external force requires a long amount of time to overcome the hydrodynamic drag acting on the particles. This limitation has been solved in passive techniques where separation is achieved through the only action of fluid inertia [21,22]. 

One of the advantages of passive techniques is the possibility to integrate a microfluidic sorting system into miniaturized devices, known as Lab-on-Chip systems (LoCs). The growth of LoC since the 1970s, driven by the development of a Micro-Electro-Mechanical-System (MEMS), has met these requirements. LoCs are miniaturized microfluidic devices, typically a few square centimeters in size, capable of manipulating small volumes of fluids through the use of micrometer-sized channels, pumps, and tubes, allowing rapid diagnostics and therapy. George Whitesides, a pioneer of microfluidics, defined it as “the science and technology of systems that manipulate small quantities of fluids through channels 10 to 100 μm in size” [23].

Microfluidic devices, therefore, represent a promising technology for point-of-care testing and diagnosis, adhering to the criteria recommended by the World Health Organization (WHO) to be Affordable, Sensitive, Specific, User-friendly, Rapid and Robust, Equipment-free and Delivered to those who need it (ASSURED) [24].

Among passive techniques, the main ones are pinched flow fractionation (PFF) [25], deterministic lateral displacement (DLD) [26], hydrophoretic filtration [27], size exclusion filtration [28], cross-flow filtration [29], and inertial focusing [30,31].

Pinched flow fractionation involves injecting a flow containing cells into a narrow channel, which expands into a wider chamber, forcing cells to align at a distance from the channel wall depending on their size: smaller cells align closer to the wall, while larger cells remain further away [25]. On the other hand, deterministic lateral displacement utilizes a periodic array of micro-obstacles within the channel of the device, directing smaller particles in one direction and larger particles in another, depending on the placement of the obstacles [26]. Hydrophoretic filtration, similar to DLD, utilizes a periodic array of step-like obstacles inside the channel, creating a pressure gradient that directs particles of lower density to different zones than particles of higher density [27]. In the size exclusion filtration method, a columnar micro-obstacle pattern allows for the selection of cell groups based on their size and shape [28]. The cross-flow filtration technique, however, employs a membrane with pores that retain larger size particles while allowing smaller ones to pass through [31]. 

Inertial focusing techniques exploit the action of inertial forces acting on particles suspended in a fluid within a microchannel. These techniques are based on inertial effects that occur between the Stokes flow regime (laminar) and turbulent flow regime [32]. Inertial and viscous forces act on particles in fluid confined within a micrometer channel, causing them to migrate towards specific equilibrium positions. The dimensions and geometry of both channel and particles play a critical role in this effect, resulting in lateral migration in straight channels [31,33] and the generation of Dean secondary flows in curved channels (i.e., serpentine, spiral channels) [34,35,36,37,38]. Additionally, the microchannel cross-sectional shape influences how inertial and viscous forces balance, thereby affecting particle behavior. 

Inertial particle sorting using spiral microchannels with different cross-sections requires further numerical simulations and experimental studies to fully understand how different experimental conditions, such as channel dimensions, cells type, channel cross-sectional shape, affect particle behavior. Therefore, a well-organized and clear review of the existing literature is essential to guide future research in this field.

This review aims to provide a comprehensive overview of the developments and advantages of inertial focusing in spiral microchannels used for cell sorting. We begin by discussing the inertial sorting physical phenomenon in straight micrometric channels, followed by an exploration of how curvature in channel geometry enhances particle migration to achieve more stable equilibrium positions. We subsequently present common materials and methods used for spiral inertial-based Lab-on-Chip (LoC) fabrication and testing. An in-depth survey of geometries explored in the literature will focus specifically on how cross-section channel shapes influence the sorting efficiency based on the sizes of the particles to be separated. Various successful outcomes have been achieved in sorting different types of particles (CTCs, bacteria, viruses, blood cells) by modifying the cross-sectional geometry. 

## 2. Theoretical Background: Inertial Focusing

Lateral migration was first studied in 1960 by Segrè and Silberberg [39]. Their experiment consisted in the observation of neutrally buoyant microscopic polymethylmethacrylate spherical particles flowing in a viscous fluid; a neutrally buoyant particle is one that neither sinks nor floats when placed in a fluid, whose density matches the density of the particle. In this state, the gravitational force is balanced by the buoyant force keeping the particle suspended at a constant depth or position within the fluid.

**Figure 1:**
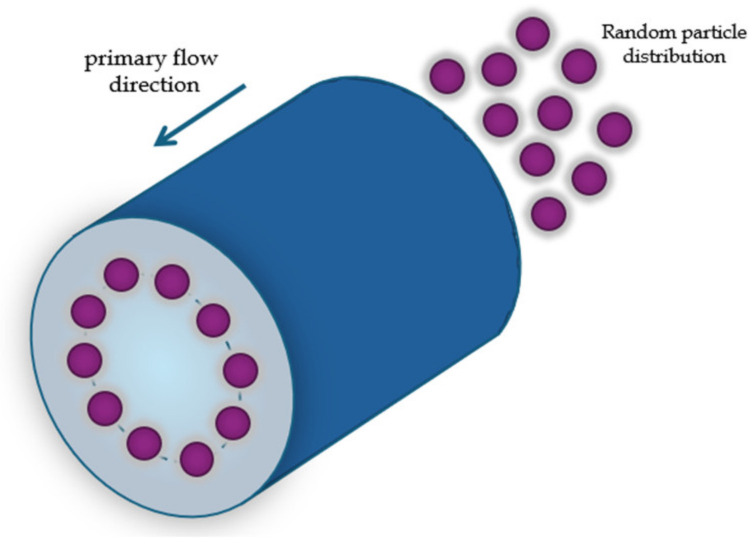
Schematic representation of the “tubular pinch effect”.

In their experiment, they observed that the spheres, when flowing in a viscous fluid through a straight tube with a circular section and a hydraulic diameter $D_{h}=1cm$, positioned themselves in a circular arrangement within the tube section at a distance of $0.2\cdot D_{h}\;$ from the tube walls (Figure 1); the hydraulic diameter is defined as [40]:

(1) $$D_{h}=\frac{4\times cross\;sectional\;area}{cross\;sectional\;perimeter}\;\;$$

which, for a circular channel, equals the diameter of the circular cross-section.

This phenomenon was named the “tubular pinch effect”. Segrè and Silbeberg also suggested that this effect could have applications in the fractionation of particles of different sizes [39].

This effect has been extensively studied over the years in order to better understand the behavior of particles flowing in a tube [33]. Particle behavior in the fluid depends on the flow regime, which is characterized by the Reynolds number $R_{e}$, a dimensionless number defined as the ratio of inertial forces to viscous forces, calculated as:

(2) $$R_{e}=\rho \;U_{f}D_{h}/\mu \;$$

with ρ  the fluid density, $U_{f}$ the average velocity of the fluid and μ  the dynamic viscosity. As expected, in microfluidic devices, $R_{e}$ is typically very low (between $10^{-6}$ and 10), so inertial effects can be neglected. However, in microchannels, where a laminar flow regime is established with $R_{e}<2000$, inertial effects become significant [40,41].

In straight microfluidic tubes, for moderate Reynolds numbers (${1<R}_{e}<10^{2}$), inertial forces begin to dominate over viscous forces. Under these $R_{e}$ conditions, the flow is laminar and not turbulent and is called Poiseuille flow. When fluid is confined by channel walls, the velocity profile of the fluid is parabolic because the walls create friction that slow the fluid’s streamlines. The fluid velocity gradient induces the particle to be retarded in both perpendicular and parallel directions with respect to the walls due to drag caused by the walls, resulting in the emergence of the Magnus lift force, Saffman force and wall-lift force [10,32].

The Magnus lift force is a consequence of particle rotation due to the fluid’s velocity gradient around the particle and acts perpendicular to both the rotation axis and the direction of particle’s motion [32]. The rotation induces fluid circulation around the particle: the fluid velocity on the upper part of the particle increases, causing streamlines to be closer together, and the pressure is low, while the fluid velocity on the lower part of the particle decreases, spreading the streamlines apart and increasing pressure. Then, as a result of this transverse pressure gradient, a lift force is generated caused by the asymmetry in the flow streamline [40]. 

The drag caused by walls exerts a lateral force on the particle known as the Saffman force. This force arises from the interaction between the particle’s velocity field and the fluid velocity gradient, and it acts towards the channel wall. The Saffman force is defined as:

(3) $$F_{s}=\frac{K}{4}V\;a^{2}{\left(\gamma \;v^{-1}\right)}^{\frac{1}{2}}$$

where K is a constant, V is the relative velocity between the particles and the fluid, a the particle diameter, γ is the velocity gradient, and v the kinetic viscosity. For a neutrally buoyant particles in Poiseuille flow, no Saffman force acts on the particles. 

**Figure 2:**
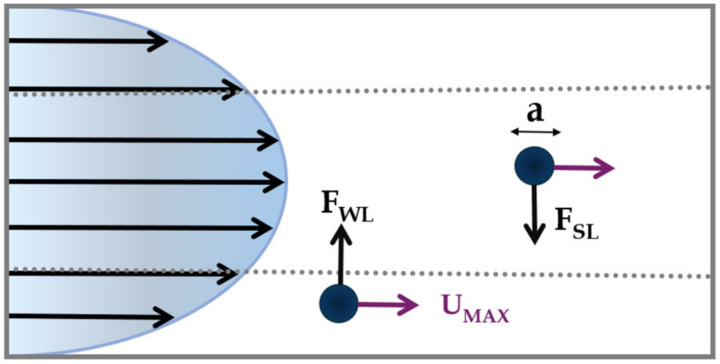
Balancing of wall-lift force and shear-lift force acting on particle in a rectilinear channel.

As mentioned before, the presence of walls modifies the flow field around the particle, causing its motion to be retarded in both the parallel and perpendicular directions with respect to the primary flow [10]. A lateral migration of the particle emerges as the consequence of two forces perpendicular to the primary fluid flow: (i) the $F_{SL}$ shear-lift force, and (ii) the $F_{WL}$ wall-lift force (Figure 2). 

The first force, the shear-lift force, acts radially from the center towards the channel walls and arises from the parabolic velocity profile of the fluid. It is defined as:

(4) $$F_{SL}=\frac{f_{SL}\;\rho \;U_{max}^{2}a^{3}}{D_{h}}$$

where $f_{SL}$ is the shear-lift coefficient, ρ is the fluid density, $U_{max}$ is the maximum fluid velocity, a is the particle diameter and $D_{h}$ is the channel hydraulic diameter. For a circular channel, $D_{h}$ equals the diameter of the circular cross-section while for a rectangular channel, it is defined as $D_{h}=2wh\;/(w+h)$, where w is the width and h the height of the cross-section.

The second force, the wall-lift force, arises from the interaction between particles and the tube walls. It is directed towards the tube center [42] and is defined as:

(5) $$F_{WL}=\frac{f_{WL}\;\rho \;U_{max}^{2}a^{6}}{D_{h}}$$

where $f_{WL}$ is the wall-lift coefficient.

The balance between $F_{SL}$ and $F_{WL}$ defines the net inertial force, derived by Asmolov [43]:

(6) $$F_{L}=f_{L}\frac{\rho U_{max}^{2}a^{4}}{D_{h}}$$

where $f_{L}$ is the lift coefficient, which is a function of both the lateral particle position and the Reynolds number $R_{e}$ ($f_{L}\propto \frac{H^{2}}{a^{2}}\sqrt{Re}).$ Under this force, a small spherical particle with $\frac{a}{D_{h}}\ll 1$ [43] migrates towards distinct equilibrium positions across the streamlines. The net inertial force acting on particles then, has a biquadratic dependence on particle size ($F_{L}\propto a^{4}$) and on geometric properties of the channel.

The particles’ Reynolds number $R_{p}$, which is defined as:

(7) $$R_{p}=R_{e}{\left(\frac{a}{D_{h}}\right)}^{2}=\frac{\rho U_{f}}{\mu \;D_{h}}.$$

This takes into account the size ratio of the particle to the channel and can be used to predict the particle’s fluid dynamic behavior. For $R_{p}$ on the order of 1, the inertial lift force becomes dominant, leading particles to migrate towards distinct equilibrium positions. Conversely, when $R_{p}\ll 1$, viscous drag dominates, and particles tend to follow the fluid streamlines [44].

First, in 2007 Di Carlo et al. [34], theoretically predicted and demonstrated trough experiments, using particles of different sizes, that for particles to reach the equilibrium position the ratio $a/D_{h}\;$ had to be greater than 0.07. The same result was later experimentally confirmed by [45]. 

As discussed, the hydraulic diameter ($D_{h})$ influences the particle behavior and, consequently, the width and the height of the channel. Another important criterion is the aspect ratio (AR) of the microfluidic channel, defined as the ratio between its width and height. 

In circular cross-section channels, particles migrate to form an annular pattern [39,46]. In square cross-section channels with an aspect ratio AR of 1, four equilibrium positions are observed at the center of each channel walls [34]. In rectangular section channels, where the aspect ratio AR is greater or less than 1, the number of equilibrium positions reduces from four to two, with particles locating at the midpoint of each longer channel walls [31]. 

In systems with particles of various sizes, flowing in rectilinear channels, a low value of the ratio (AR≈0.5) makes larger particle to reach their focusing equilibrium positions more quickly, while smaller ones require a longer distance to reach their equilibrium position. The minimum required length ($L_{MIN}$) for particles to achieve focusing can be estimated by [47]:

(8) $$L_{MIN}=\frac{3\pi \;\mu \;D_{h}^{3}}{\rho \;U\;a^{3}}.$$

The equation shows a cubic dependence on $1/(\frac{a^{3}}{L_{c}^{3}})$, where $L_{c}$ is the characteristic length the microchannel; for square cross-sectional microchannels, $L_{c}$ is equal to the microchannel hydraulic diameter $D_{h}$, while for rectangular cross-sectional microchannels, it is equal to the narrowest dimension of the channel [47].

Despite the ease of fabrication and operation of straight channels, designing microchannels with a sufficient side length $L_{MIN}$ to allow particle focus on equilibrium positions implies higher flow resistance and a large device footprint.

The inefficiency in separating cells of different sizes in straight microchannels has led to the design of curvilinear microchannels with a low aspect ratio (AR<1) [10]. Curvilinear microchannels include serpentine or spiral geometries; while serpentine channels have alternating curvature, spiral channels have a curvature along a single direction. 

### 2.1. Curvilinear Microchannel and Dean Vortices

**Figure 3:**
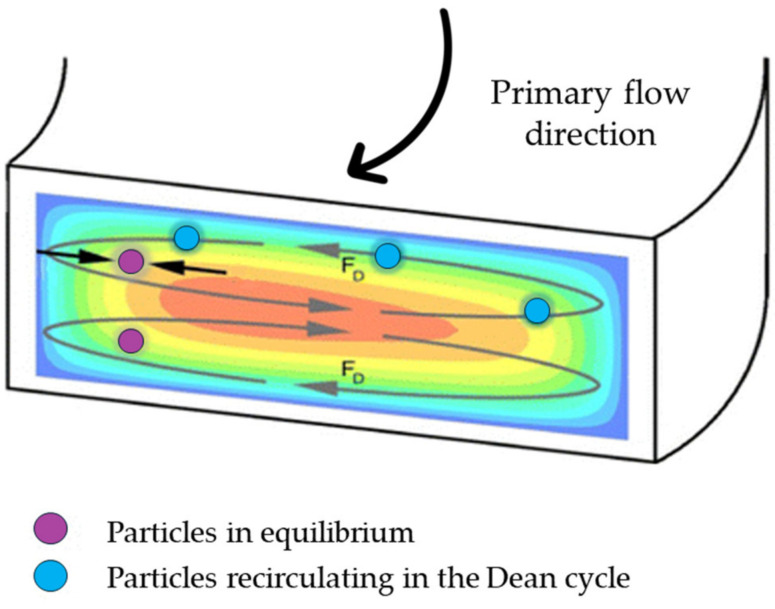
Schematic representation of secondary flow and Dean vortices.

Introducing a curvature with a radius $R_{c}\;$ in the channel geometry, a secondary flow, known as Dean flow, arises due to the flow velocity difference in the downstream direction between fluid in the central and fluid near the walls of the channel [48]. Fluid elements around the center of the tube move in circular motions, creating a radial pressure gradient directed towards the upper and lower walls of the tube. This pressure gradient leads to the formation of two symmetrical vortices perpendicular to the main flow direction (Figure 3).

The distribution and strength of these vortices are related to a dimensionless parameter, the Dean’s number (De):

(9) $$De=R_{e}\sqrt{\frac{D_{h}}{2r_{c}}}$$

where $R_{e}$ is the Reynolds number, $D_{h}$ the hydraulic diameter of the channel and $r_{c}$ is the radius of curvature of the convex surface of the curvilinear channel. 

Hence, particles in a curvilinear channel experience both the net inertial force $F_{L}$ and the drug force:

(10) $$F_{Dean}=3\pi \;\mu \;a\;U_{D}$$

where $U_{D\;}=1.8\cdot 10^{-4}\;De^{1.63}\;$ is the Dean velocity, i.e., the lateral migration velocity of the particles [49].

The competition between the net inertial force $F_{L}$ and the drug force $F_{Dean}\;$ determines the migration of particles towards equilibrium positions in a curvilinear microchannel. It must be noted that the Dean force does not contribute to creating new focusing positions; rather, it acts additionally to inertial forces to reduce the number of equilibrium positions. Consequently, particles migrating to different locations become unstable and return to initial equilibrium positions [34]. 

Along a Dean vortex, a particle traverses a lateral distance traversed defined as “Dean cycle”; under a Dean cycle, a particle migrates from one channel wall to the opposite wall and then returns to its initial position. The length of one complete Dean cycle migration can be approximated as $L_{DC}\approx 2w+h$, where w is the microchannel width and h is the microchannel height [50]. Thus, the total microchannel length required for a particle to reach Dean migration is given by $L_{C}=\frac{U_{F}}{U_{D}}L_{DC}$.

In ideal conditions, Dean drag force is of the same order as the net inertial lift, leading particles to reach the lift-induced equilibrium positions while interacting weakly with the Dean flow. If the inertial lift force ($F_{L}$) is greater than the Dean drag force ($F_{Dean}$), then the focusing mechanism is continually interrupted. Conversely, if $F_{L}$ is weaker than $F_{Dean}$ focusing will be only due to inertial lift. As suggested by [34], the ratio of lift force to the drag force scales as:

(11) $$\frac{F_{L}}{F_{Dean}}\sim {\left(\frac{D_{h}}{2r_{c}}\right)}^{-1}{\left(\frac{a}{D_{h}}\right)}^{3}R_{e}.$$

From this, it can be noted that as $D_{h}/2r_{c}$ and the Dean Number (De) approach 0 (i.e., in straight channels), the Dean drag force dominates over the net inertial force causing the particles to remain confined within the Dean vortices. The cubic dependence on the ratio of particle to channel dimensions suggests that smaller particles may not focus, whereas larger particles focus more quickly. Conversely, as $D_{h}/2r_{c}$ and De approach ∞, the net inertial force dominates over the Dean force. In this case, the mechanism is similar to that observed in straight channels, where all particles will be defocused.

Therefore, the dependence on the hydraulic diameter implies that as channel dimensions increase, particles experience weaker inertial forces and stronger Dean drag forces [51].

The dependence of the Dean number on the radius of curvature ($r_{c})\;$ highlights the importance of the type of spiral chosen. One of the most studied spirals in this regard is the Archimedean spiral; this can be defined as a curve where each loop is spaced from the next one by a constant amount. The constant increase in the radius of curvature has been shown to induce a gradual development of vortices due to the secondary flow, thus affecting the minimum focusing length [35]. The number of loops in the Archimedean spiral is correlated with the channel length; therefore, it is essential to determine the appropriate number of loops to ensure that the particles reach and focus on their equilibrium positions. 

### 2.2. Rectangular and Trapezoidal Spiral Microchannels

As explained in Section 2, the cross-sectional geometry of the channel plays a significant role in particle migration towards equilibrium positions in spiral microchannels. 

Different studies have been conducted to understand the focusing mechanisms in spiral channels with square [52], rectangular [53], trapezoidal [54], hybrid cross-sectional shapes [55]. 

**Figure 4:**
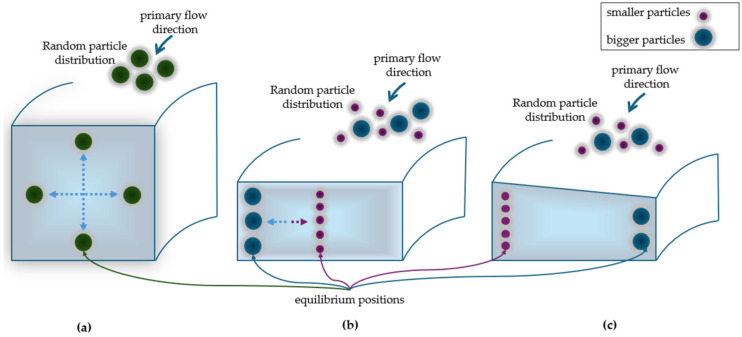
Equilibrium positions of particles in (**a**) squared, (**b**) rectangular and (**c**) trapezoidal cross-section channels.

In a square channel, particles migrate to four equilibrium positions, typically located near the corners or at the center of each edge of the channel (Figure 4a) [52]. 

In a rectangular section channel, the interplay between inertial lift force and Dean drag force leads to the formation of symmetrical Dean vortices in the upper and lower sections of the channel. This interaction causes particles to migrate across the channel width until they reach their equilibrium position near the inner or outer wall. In case of mixed particles sizes, smaller particles tend to focus towards the center of the channel, while larger particles will focus nearby the outer wall (Figure 4b).

In a trapezoidal section channel, the asymmetry in the channel geometry affects the formation of Dean vortices, as demonstrated by Guan et al. [53]. By varying the heights of the inner and the outer walls, two asymmetric Dean vortices appear, causing smaller particles to be directed towards the outer half of the channel wall, while the larger ones towards the inner half of the wall. This creates stronger vortices and a more distinct separation between the equilibrium positions [56], improving resolution for particle sorting (Figure 4c). The use of trapezoidal-section spiral channels was first studied by Wu’s researchers’ group [57]. Since the publication of this work, various studies have demonstrated the effectiveness of this design in separating different particles type, including the isolation of leukocytes from circulating tumor cells (CTCs) in a blood samples [54], the selective separation of microalgae cells [58] and the identification of the bacteria responsible for the deterioration of beer [1]. In addition to rectangular, trapezoidal and triangular geometries, studies have been conducted to improve the separation efficiency of the devices designing hybrid cross-section channels, by combining trapezoidal and rectangular geometries. These specific studies will be discussed in Section 4.4. [59]. 

## 3. Materials and Prototyping Techniques for Spiral Microchannels 

As explained, Archimedean spiral microfluidics represent an interesting design for the prototyping of miniaturized LoCs for cell sorting, thanks to their extended channel length, which allows particles to focus on their equilibrium positions. The fabrication process for such channels with different cross-sectional geometries (squared, rectangular, trapezoidal or complex) must match with the replication process’s speed and simplicity to be industrially scalable.

Then, selecting the appropriate prototyping technique in addition to the choice of material for the LoCs fabrication is crucial. Many studies have been published about general microfabrication techniques and materials for microfluidics, such as [60,61,62,63,64].

In the following paragraphs, we will present the most commonly used materials for LoCs production, highlighting their limitations and advantages for fabricating spiral inertial microfluidic chips. Moreover, techniques for producing rectangular, trapezoidal and complex cross-sectional spiral microchannels will be outlined. A review of the most suitable fabrication techniques is essential, since the fabrication of slanted or complex spiral channels can be very expensive due to the challenges associated with conventional micromachining methods [38]. All relevant details from the studies discussed in these sections will be summarized in Table 1 and Table 2 to provide the reader with a quick overview [36,65].

### 3.1. Materials

The main used materials for LoCs fabrication can be divided into two categories: (i) inorganic materials (e.g., metals, ceramics, and glasses) and (ii) organic materials (e.g., polymers and biodegradable materials).

Metals, such as iron, aluminum, copper, and their alloys, exhibit desirable properties such as cost-effectiveness, high availability, ease of processing, and good resistance to high temperatures and pressures. However, they may pose challenges when interacting with biological fluids. Although noble metals, such as gold or platinum, could enhance biocompatibility, disadvantages such as high costs and lack of optical transparency make them not ideal for these applications [60]. For this reason, no metallic spiral microfluidic devices for biological liquids and cells manipulation have been reported. 

Apart from metals, commonly used inorganic materials for LoC production include glass, silicon, and ceramics. Silicon has been widely used in recent decades for its availability, chemical compatibility, thermal stability, and the ability to fabricate devices with nanometer resolution. However, silicon’s opacity to visible and UV radiation makes it unsuitable for direct real-time imaging, which is required for cell sorting monitoring and for fluorescent optical detection. Moreover, its poor mechanical properties, such as fragility and high elastic modulus can cause issues when exposed to high-pressure flows. The high fabrication costs make silicon an inappropriate candidate for low-cost portable Lab-on-Chip systems. Additionally, due to its lack of gas permeability it is also not suitable for long-term cell culture. For these reasons, silicon has been rarely used in microfluidics; the only reported study of a silicon spiral device fabrication for cell sorting was conducted by Gregoratto et al. [66].

Similarly, glass, which is chemically inert, biologically compatible and offers properties like thermal stability and insulation, can be a valuable option under various operational conditions. Compared to silicon, glass offers advantages such as optical transparency and lower costs but prototyping of microfluidic structures in glass is time consuming and not suitable for low-cost mass-scale devices fabrication. Ceramics, despite their good corrosion resistance and thermal stability, pose challenges due to their fragility and high porosity. As silicon, due to opacity to visible radiation, real-time imaging is an issue in ceramics-based microfluidic devices. 

Inorganic materials are characterized by poor gas permeability, which is a significant drawback for applications requiring long-term cells manipulation, as in the inertial sorting of cells and viable organism, like bacteria, algae and yeast [62]. 

Polymeric materials are widely used for microfluidic devices fabrication, especially on a large industrial scale, due to their ease and cost-effectiveness of processing. Moreover, thanks to biocompatibility and optical transparency (or semi-transparency) in the visible range, polymers are excellent candidates for cell sorting applications. Polymers are categorized as thermosets and thermoplastics [67]. The most used polymers include polydimethylsiloxane (PDMS), fluoropolymers (TEFLON, PTFE), and polymethylmethacrylate (PMMA) and UV-curable materials (epoxy resins, SU-8 photoresist) [68].

Thermoset polymers consist of macromolecules that undergo an irreversible curing process, when exposed to by heat, chemical reactions, or radiations (UV), resulting in a cross-linked network structure. The cross-linking process enhance the formation of covalent bonds between polymer chains, preventing polymers remelting or reshaping. Main characteristics of thermoset polymers include thermal stability, chemical resistance, high mechanical strength, despite non-recyclability.

Most used thermoset polymers in microfluidic devices are PDMS and resins materials. 

PDMS is commonly used in microfluidic devices thanks to its hydrophobicity, gas permeability, optical transparency, biocompatibility, and high elasticity [69]. These properties allow for the development of 3D structures and for the integration of microvalves and micropumps for fluid manipulation. However, PDMS also has several disadvantages that impact its use in microfluidics. These include channel deformation due its high mechanical compliance, leaching-out of uncrosslinked oligomers evaporation and adsorption and hydrophobic recovery over time after hydrophilic treatment [63]. Mechanical deformation is particularly problematic in cell cultures and fluids manipulation due to subjection to high pressures while its high oxygen permeability can cause a hyperoxic environment, leading to cellular stress. 

Despite these aspects, PDMS remains the most widely used polymer for spiral microchannel fabrication for cell sorting in combination with the soft-lithography technique that will be explained later. 

Epoxy resins consist of epoxy groups that undergo strong crosslinking when exposed to UV light, resulting in excellent mechanical, thermal, and chemical stability. SU-8 photoresist belongs to this class of materials and can be patterned into nanometric-to-millimetric structures through both photolithography and laser ablation techniques [70]. 

Thermoplastic polymers are classified based on their capability to soften and become malleable when exposed to heat, and then solidify upon cooling in a reversible process. This property arises from their linear or branched polymer structures, which lack permanent cross-linking between chains. As a result, thermoplastics can undergo multiple cycles of heating and cooling, making them recyclable. Beyond this, thermoplastic polymers gained popularity due to their chemical resistance, optical transparency, and solvent compatibility. An example of thermoplastic polymer is polymethylmethacrylate (PMMA). PMMA, is used due to its low production costs and its recyclability after decomposition at temperatures above the glass transition temperature (T_g_), making it ideal for sustainable “green microchips” fabrication [71]. It exhibits high rigidity, which is optimal for applications requiring high pressures, optical transparency in the visible range, excellent mechanical and electrical properties, solvent compatibility (except with organic solvents and hydrocarbons). Even if it is still less used than PDMS, PMMA represents an optimal candidate for rigid spiral microchannel devices allowing for f high operating flow rates without leakage [2,72].

The last category includes biodegradable materials, such as paper, hydrogels and wax. Paper has become an interesting material for chip fabrication due to its numerous properties like low costs, bio-affinity, accessibility, lightweight and ease of fabrication; however, it lacks mechanical resistance when exposed to liquids and transparency. Hydrogels, which are three-dimensional structures of hydrophilic polymer chains, allow the diffusion of bioparticles and small molecules, presenting high biocompatibility, biodegradability, and low toxicity. However, the difficulty in maintaining device integrity and channel geometry in hydrogels under different operational conditions, such as high-pressure flows required for inertial focusing in micrometer channels, makes them a challenge [73,74,75].

The main advantages and disadvantages of the presented material are provided in Table 1.

**Table 1:** Principal advantaged and disadvantages of materials used for spiral microchannels [60,62,76].

Material	Advantages	Disadvantages	Ref.
PMMA	Low production costs; recyclability; disposable use; good biocompatibility; chemical inertia; optical transparency for real-time imaging.	Medium gas permeability.	[2,72,77]
PDMS	Good biocompatibility; disposable use; chemical inertia.	No recyclability; medium production costs.	[1,35,45,50,53,54,55,57,58,59,78,79,80,81,82,83,84,85]
Glass	Good biocompatibility; disposable use; chemical inertia; optical transparency for real-time imaging.	From low to moderate gas permeability.	[49,86]
Silicon	Recyclability.	High production costs; no disposable use; no transparency for real-time imaging.	[66]
Resin	Biocompatibility; medium optical transparency for real-time imaging.	Medium biocompatibility, high production costs; difficult disposable use.	[87]
Hydrogel	High biocompatibility.	No recyclability; from medium to high production costs; difficult disposable use.	[74]

### 3.2. Fabrication Techniques

In a study published in 2011, Waldbaur et al. [60] classified the fabrication techniques for microfluidic devices into two main categories based on whether the microchannels structure is replicated or not: (i) by deposition of material (replication techniques via mold) or (ii) by direct material removal. In the text that follows, we have chosen to use this categorization to review the techniques employed for the fabrication of spiral inertial chips [36]. Table 2 outlines the key advantages and disadvantages of the following fabrication techniques. 

#### 3.2.1. Techniques by Deposition

Soft lithography is the most used technique for the fabrication of microfluidic devices, including spiral microchannel structures on PDMS substrates. It consists of creating a hard master (often in silicon), pouring liquid polymer (PDMS) into the mold, heat curing it, then subjecting it to a room temperature hardening process; once the polymer has cured, the hardened polymer substrate is peeled off and removed. Soft lithography provides high-resolution replicas and allows for the fabrication of three-dimensional geometries with low costs and rapid production [64]. Disadvantages of this technique are related to the replica mold and to logistic requirements. First, due to the softness of the material used, deformation of the pattern may occur when the removing the cast from the mold [88], so then soft lithography requires a cleanroom environment, which increases the overall process costs. 

Most spiral microchannel devices reported in the literature are fabricated using soft lithography. These include both rectangular cross-section channels with dimensions ranging from 100 to 500 µm in width and 50 to170 µm in height [35,45,50,53,78,79,82,84,85] and trapezoidal cross-section channels with dimensions ranging from 200 to 600 µm in width and 40 to 130 µm in height [1,54,55,57,58,80,83,87]. Trapezoidal cross-section shapes are more challenging to reproduce than rectangular cross-sections, especially using soft lithography, which is limited by the precision of the milling machine in creating the trapezoidal structure on the mold. 

The techniques by deposition also include electron-beam lithography, LIGA (lithography-galvanoforming), photolithography, X-ray or laser lithography and xurography [60,61]. Xurography has been used to replicate a rectangular cross-section spiral structure with a variable width from 200 µm at the inlet to 600 µm at the outlet and a height of 100 µm on a PDMS substrate [89]. This technique involves creating a mold, cutting the design with a plotter, covering it with PDMS and using a microwave oven for curing.

These techniques come with certain limitations: (i) they require an additive process to fabricate the patterned mold or the hard master; (ii) molds used are usually made from soft materials, causing deformation of the channel geometry when removing the cast from the mold; and (iii) semi-cleanroom operational conditions are necessary [64]. These aspects increase the costs of such lithography techniques.

3D-printing is an additive manufacturing technique that involves creating three-dimensional structures layer by layer from a CAD model by depositing fused material. Laser selective sintering in a resin bath, powder bed fusion, or inkjet 3D printing are a few examples of additive manufacturing techniques used for the fabrication of micro-devices [60]. The potential of applying 3D-printing to challenging materials as hydrogels has been investigated also by Shen et al.; the group fabricated different microfluidic designs, including a spiral microchannel, using a hydrosoluble and photo-crosslinkable chitosan methacryloy (CS-MA) [22]. While 3D printing allows for the production of microfluidic designs with different cross-sections, this technique has limitations in z-resolution during layer-by-layer deposition, especially when structuring hollow channels and voids, which prevents a precise reproduction of the device’s geometry. Additionally, some materials used in 3D-printing (such as polymers, inorganic materials, metals or hydrogels) are not transparent, making the devices not suitable for real-time imaging. Bazaz et al. [87] utilized 3D printing to prototype a resin (BV-007) spiral microchannel with a triangular cross-section of 600 µm in width and 210 µm in height, underlining the flexibility of this method for creating complex cross-section. Raoufi’s group used 3D-printing to create a wax mold of a spiral microchannel with complex cross-section (hydraulic diameter of 250 µm), by depositing molten wax droplets layer by layer. The 3D-printed wax mold was poured with liquid PDMS and curing agent, followed by heat curing.

#### 3.2.2. Techniques by Removal 

Etching involves removing material to create the microfluidic structure via solvent (wet etching) or via plasma or particle beams (dry etching). Although etching allows for high processing speeds, wet etching requires the use of corrosive solvents, which introduces significant safety risks. Dry etching, while safer, involves longer production times and is therefore rarely used. Etching is one of the primary methods for structuring materials such as silicon and glass. An example of application of this technique in spiral microfluidics is only in Gregoratto et al.’s study [66] on silicon substrates. They used the Bosh process to obtain a spiral channel with cross-sectional dimensions of 100 µm in width and 1250 µm in height. However, they encountered several challenges related to the thickness of the silicon wafer, resulting in difficulties in cooling it; additionally, the chemistry used during the etching process caused pocked sidewalls. 

Mechanical structuring is based on milling, planning or drilling; they rely on the material structuring using a CNC (Computer Numerical Control) process that transfers the microfluidic structure from a CAD (Computer-Aided Design) model to a machine that removes material using rotating (e.g., milling) or non-rotating cutting tool (e.g., planning). Mechanical micromilling is still widely used due to its cost-effectiveness, simplicity, process efficiency and rapidity. In spiral microfluidics, it is used for prototyping the microchannel geometry for the mask in lithography techniques [8,57]. For example, Ghadami et al. [59] used micromachining to fabricate a master mold for prototyping a PDMS stair-like microchannel (with dimensions of 500 µm in width and 110 µm in height).

Finally, pulsed laser ablation, is an effective method for generating multiple microstructures on a variety of materials, although it requires expensive equipment. This process involves scanning a laser beam over the surface according to the CAD design, removing material. As the laser beam interacts with the surface, an absorption process occurs, leading to an increase in temperature. Depending on the duration of the laser pulse, thermal degradation induces fusion (for long or short laser pulses) and/or evaporation (for ultrashort pico/femtoseconds laser pulses) of the material. Thanks to the high peak intensities and scanning speeds, pulsed laser ablation offers the advantage of creating microstructure in a few minutes but could result in deposition of re-solidified material debris around the ablated area, compromising the quality and profile of the microchannels [90]. These drawbacks are eliminated using ultrashort pulses [91,92,93]. Ultrashort pulsed lasers allow three-dimensional microstructuring on transparent substrates at the wavelength of the laser radiation due to non-linear absorption mechanisms. 

For spiral microfluidics, CO_2_ laser has been used for PMMA surface ablation of trapezoidal cross-section through a grey-scale method [72] and for U/W-shaped cross-section channels [2]. Adel’s group used a grey-scale approach modulating the intensity and the power of the laser based on the RBG values of an image. This method allowed for precise control of the laser ablation process to create the trapezoidal cross-section geometry with a width of 600 µm, an inner height of 70 and an outer height of 110 µm. Abdel-Mawgood’s approach consisted of creating the microchannel by a single scan of a defocused laser beam to create a U-shaped cross-section, and a double scan for the W-shaped cross-section. Despite the rapidity of this process, the obtained channels lacked precise dimensions (approximately 220 µm in width and from 162/175 to 210 µm in height) and had high uncertainty due to short laser pulses [2]. 

Instead, a Yb:KGW solid-state femtosecond laser was used by Al-Halhouli’s group on glass, which allowed the structuring of trapezoidal cross-section trapezoidal channels (width of 220 µm, outer height of 60 µm and inner height of 40 µm) with smooth surface channel walls [49,86]

A key advantage of the laser direct writing technique is that it does not need a photomask and a clean room environment, reducing both fabrication time and the cost of producing microfluidic chip.

**Table 2:** Principal advantaged and disadvantages of fabrication techniques for spiral microchannels [60,76].

Fabrication Technique	Advantages	Disadvantages	Ref.
CO_2_ laser ablation	Wide range of materials; from medium to high microchannel resolution; low production costs; low production time; good scalability.	Serial process.	[2,72,77]
Yb:KGW solid-state laser ablation	Wide range of materials; high microchannel resolution; low production costs; low production time; good scalability.	Serial process.	[49,86]
Soft lithography	High microchannel resolution; low production costs; low production time; wide range of materials; good scalability.	Suitable only for photoresist and polymers; high costs for the mask.	[1,35,45,50,53,54,55,57,58,59,78,79,80,81,82,83,84,85]
Xurography	High microchannel resolution; low production costs; high production time; absence of the mask.	Suitable only for photoresist and polymers.	[89]
Etching	Wide range of materials; high microchannel resolution; low production costs; medium production time.	Use of hazardous chemicals; expensive equipment.	[66]
3D-printing	low production costs; high production time; absence of the mask.	Low microchannel resolution; Suitable only for photoresist, polymers and hydrogels.	[74,87]

## 4. Overview on Different Cross-Sectional Shapes

Due to the multiple advantageous features of microfluidic structures such as their compactness and high separation efficiency thanks to Dean’s secondary flow, scientists have shown interest in developing spiral microfluidic devices. Since the publication of the Di Carlo et al. [34] scientific report on particle focusing in curved microchannels, numerous experiments have been conducted to investigate these microfluidic structures. 

The main purpose of these studies has been to enhance the efficiency of target particle separation, defined as [55,94,95]:

(12) $$SE\%=\frac{N_{t,o}}{\left(N_{t,i}\right)}\cdot 100$$

where $N_{t,o}$ is the number of target particles at one outlet, $N_{t,i}$ is total number of particles in the channel inlet. 

It should be noted that the separation efficiency value refers to the sample introduced into the device. Frequently, the samples of raw biological fluid are subjected to pre-treatment steps before being injected into the device. These pre-treatments typically involve the pre-enrichment of the solution’s concentration to improve discrimination, reduce cell sorting time, and increase separation efficiency [96]. One of the most used pre-treatment methods is erythrocyte lysis, which involves using a buffer solution (e.g., ammonium chloride solution) to break down the RBC membranes and remove them from the solution. This prevents interference with leukocytes, reduces the quantity of blood cells in the sample, and mitigates unwanted cell-to-cell interactions [50,57,79], thereby improving target cell purity. For example, Magalhaes’ study introduces a device that employs recirculation cycles to selectively pre-enrich low-concentration samples, enhancing the detection of target cells. Sometimes, it is also preferable to perform dilution steps on the raw biological sample. For instance, in [7,72], blood samples were diluted to reduce cell-to-cell interactions resulting from the high concentration of RBCs in the blood.

As mentioned in the previous section, the cross-sectional geometry of the channel plays a critical role in the formation of Dean’s vortices, enabling the formation of different equilibrium positions and thus facilitating particles sorting. For this reason, scientists have explored the effect of the secondary flow in various cross-sectional shapes over the years including rectangular, trapezoidal, triangular, complex/hybrid geometries.

For example, in 2018, Lee et al. [97] conducted a study demonstrating the use of a label-free commercial platform, ClearCell^®^ FX, driven by the CTChip^®^ FR chip for single-step isolation of circulating tumor cells (CTCs) from blood cells. The device employed Dean Flow Fractionation and inertial focusing in a spiral microchannel, successfully isolating CTCs (~24 µm) from blood cells (BCs, ~8–14 µm) in blood samples. Additionally, the device enabled consecutive isolation cycles for different samples. This study serves as an important example of the commercial production of spiral microchannel devices for cell sorting via inertial focusing. 

**Figure 5:**
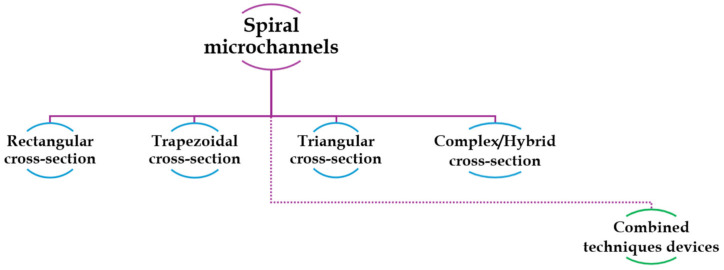
Classification spiral microchannels device with different cross-sectional geometries and combined techniques.

In the following pages, an overview of most studies of the past two decades about spiral microfluidics is provided. The studies are grouped based on the shape of the channel cross-sections, as schematized in Figure 5. An additional section, titled “Combined technique devices”, will be dedicated to studies that combine of inertial focusing in spiral microfluidic devices with other cell sorting techniques. For each geometry, in each subsection, information about the type of particles or cells investigated and optimal flow rates to maximize the sorting mechanism and the separation efficiency, are reported.

The most significant information of each cited study is also summarized in Table 3. which offers to the reader a simplified and complete overview.

### 4.1. Rectangular Cross-Section Channels

The first spiral microfluidic device was proposed in 2007 by Gregoratto’s group. The authors designed two devices with spiral geometries on a silicon substrate. Both microfluidic spiral microchannels had a rectangular cross-section with an aspect ratio (AR) of 15 but they differ in channel lengths: L_1_ = 25 cm and L_2_ = 50 cm_,_ respectively. For the L_1_ sample, four symmetric and asymmetric bifurcation with various ratios were chosen, while for the L_2_ sample, a symmetric bifurcation was employed. The group observed the formation of a single stable Dean vortex after a ¼ loop from the inlet. They noted a significant correlation between the flow rate and the particles concentration collected at each outlet: the highest ratio of inner outlet to outer outlet of particles concentration was 3.5, achieved for the L_2_ device at a flow rate of 2 mL/min. This result was attributed to the device’s longer length, which was necessary for particles to reach their focusing equilibrium positions [66].

Subsequently, Bhagat et al. [78] presented a study on a PDMS device with a 5-loop rectangular cross-section spiral microchannel. They first performed numerical simulations of the device behavior, assuming a water solution as liquid medium and polystyrene microparticles of sizes 1.9 and 7.32 µm, respectively, at different flow rates. They found that the device could be used for the separation of fluorescent microbeads at a flow rate of 10 µL/min, achieving a focusing efficiency of 100% for both particles type through the inner and the outer outlet. Consistent with the work of Di Carlo et al. [34], which indicated that microbead focusing occurs when a_p_/D_h_ ≥ 0.07, it was found that particles with diameters below a threshold value of approximately a_p_ ≈ 5 µm (a_p_/D_h_ < 0.06) were more affected by Dean forces than inertial forces, migrating towards the outer channel wall, differently from larger particles (a_p_/D_h_ > 0.07).

**Figure 6:**
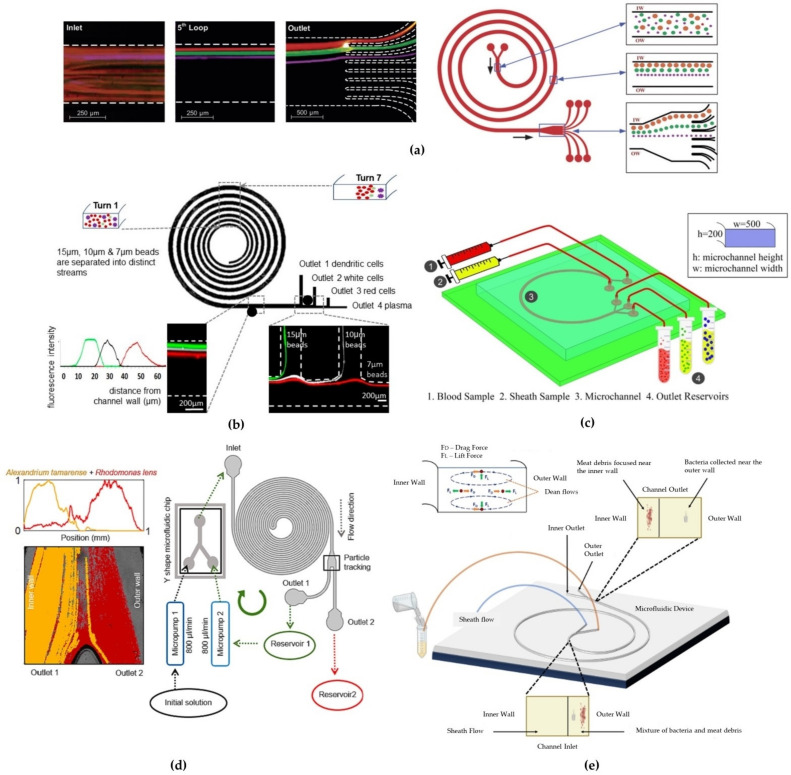
(**a**) Scheme of the spiral microchannel with particles different equilibrium positions along the inner wall (reprinted with permission from [45]). (**b**) Spiral microfluidic device with increasing channel widths and images of focused particle of three different sizes streams at the outlets (reproduced from [89] under http://creativecommons.org/licenses/by/4.0/, accessed on 1 May 2024). (**c**) Schematic diagram of the microchip device (reproduced with permission from [94]). (**d**) Design of the double-spiral and tracking representation of *Alexandrium tamarense* and *Rhodomonas lens* at the outlet region (reproduced from [83] under http://creativecommons.org/licenses/by/4.0/, accessed on 1 May 2024). (**e**) Illustration of the separation method and microchannel for sorting of Escherichia coli and Staphylococcus aureus (readapted from [85] under http://creativecommons.org/licenses/by/4.0/, accessed on 10 July 2024).

In a subsequent study from the same group [45], a device was tested for the separation of larger-sized cells. They designed a 5-loop spiral microchannel with a rectangular cross-section to separate fluorescent polystyrene microbeads with diameter of 10, 15, and 20 µm (Figure 6a), respectively. By implementing a device with eight outlets, they successfully separated different microbeads with a separation efficiency of 90% for the three particles sizes at flow rates of approximately 3 µL/min, corresponding to a Dean number of De ~ 14.4. They further tested the device by modifying the cross-section dimensions to W = 500 µm and H = 120 µm for the separation of SH-SY5Y neuroblastoma cells (a_p_ ≈ 15 µm) and C6 glioma cells (a_p_ ≈ 8 µm) at a flow rate of 3 µL/min (De ~ 13). They achieved a separation efficiency of ~80% for both the SH-SY5Y cells at outlet 1 and the C6 glioma cells at outlet 2, thanks to the large variations in the cell sizes (~5 µm), with a 90% cell recovery after 24 h.

Confirming such promising results obtained by exploiting inertial focusing in spiral microchannels, Nivedita et al. [35] proposed two devices for continuous separation of erythrocytes and leukocytes from diluted blood samples. They fabricated two spiral microchannel designs with a rectangular cross-section and different aspect ratios. The first device featured a three-outlet system while the second was designed with a four-outlet system. Testing the devices with polystyrene beads, they observed that in the first device, at the outlet bifurcation, all particles followed the flow and were collected in the inner outlet (outlet 1), thus no separation was observed. Conversely, in the second device, particles were focused into two distinct flow streams: a broad stream near the inner wall, which eluted in the first outlet, and a narrow stream, closer to the center of the channel, which eluted in the second and third outlets. This achieved a high separation efficiency (~95%) at a flow rate of 1–2 mL/min. 

Son et al. [98] suggested that a multiple trapezoidal spiral device could enable the mechanical focusing and separation of sperm cells from red blood cells (RBCs) and other debris, such as white blood cells (WBCs). Although sperm cells have an irregular shape, they were assumed to be spherical particles with a diameter of ~5 μm, while RBCs were approximated as spherical particles with a diameter of ~9 μm. At a flow rate of 0.52 mL/min, the device demonstrated the focusing of sperm cells towards the outer wall and RBCs towards the inner wall of the channel. By collecting samples from four different outlets, they achieved a separation efficiency of 81% for non-motile sperm at the outer outlets and 99% for RBCs at the inner outlets.

Warkiani’s research group, after two earlier studies on trapezoidal cross-section channels [8], published a subsequent study proposing a new rectangular design [50]. They prototyped a 3× multiplexed system consisting of two loops spirals with a rectangular section to increase throughput using asymmetric outlets. Starting from a flow rate of 100 µm/min, they observed the distribution of the cells across the channel width in the outlet region, using a microscope with a phase-contrast light source and a high-speed camera. They conducted the experiment flowing WBCs and cancer cells separately. The larger particles and cells (>15 µm) remained at their equilibrium positions near the inner wall without lateral movement. Smaller particles and cells, instead, moved from the side of the outer wall towards the inner wall, and then back near towards the outer wall, as they flow through the channel. They successfully isolated two types of lung and breast CTCs of 12 µm size from BCs, at a flow rate of 1.7 mL/min, despite the presence of some RBCs debris in the output sample, which prevented the precise calculation of the separation efficiency.

An innovative spiral structure with a rectangular cross-section was prototyped by Caffiyar [89] to separate red blood cells, white blood cells and dendritic cells from blood fluid. The microchannel had a width that expanded from 200 µm at the inlet to 600 µm at the outlet (Figure 6b). This geometry was designed to overcome the defocusing of streamlines and mixing of cells due to the balance between lift and Dean forces in narrow channel widths. Additionally, authors declared they were limited by the low precision of the lithographic technique used to fabricate narrow-width spiral channels. When testing the device both with polystyrene beads and a mixture of human dendritic cells, white blood cells and red blood cells, they reached a separation efficiency of 72% for dendritic cells with an optimal flow rate of 1.5–1.9 mL/min.

Shiriny and Bayareh [94] proposed a single-loop rectangular cross-section channel consisting of two inlets and two outlets, to provide a simpler design for industrial fabrication compared to multiple loops (Figure 6c). Mathematical simulations with bloodstream containing WBCs and RBCs along with CTCs (MCF-7 and HeLa) demonstrated an approximate separation efficiency of 100% at throughput flow rates in the range from 113 to 139 μL/min. Numerical simulations predicted that MCF-7, HeLa and blood cells would focus close to the inner wall of the channel, the central area of the channel, and near the outer wall of the channel, respectively. This allowed the three different types of cells to be separated at the three outlets. The separation efficiency of blood cells was 80%, while 100% of MCF-7 and HeLa cells exited through the other two different outlets. Regarding separation purity, which is the number of target cells over the total number of cells at a given outlet, for MCF-7 cells and blood cells, separation purity was 100%, while for HeLa cells, it was 83%, as 20% of blood cells exited through the same outlet. 

Magalhães’s group demonstrated the applicability of inertial focusing to a wide variety of cells [83]. Their study focused on the separation of algal dinoflagellate species of different size from complex seawater samples to increase cell concentration before in situ measurements. Most microalgae species, such as *Alexandrium*, *Karenia*, and *Dinophysis*, range between 20 and 60 μm in size, though a significant portion of species are characterized by smaller cell sizes, less than 20 μm (e.g., *Chlorophyta*, *Haptophyta*, *Cyanobacteria*, *Rhodomonas lens*). Using a double rectangular spiral microfluidic device, they extended the microchannel length by adding some curves to allow the desegregation of long-chain cell conglomerates (Figure 6d). They also designed an experimental configuration using a micropumps system to recirculate through different cycles the solution for both enhancing *Alexandrium tamarense* concentration and selectively collecting *Rhodomonas lens*. Cell separation at the outlets was quantified by observing fluorescence emission signal at different wavelengths for each cell type. After three cycles of circulation with a mixture of *Alexandrium tamarense* (~30 μm) and *Rhodomonas lens* (~10 μm) at a flow rate of 2000 μL/min, they observed an increased concentration of in *Alexandrium tamarense* in the outlet 1 reservoir. The recirculation device provided a way to detect low-concentration samples thanks to selective concentration enrichment before sorting them through the microfluidic channel.

The challenge of applying spiral inertial microfluidics to different cells was also demonstrated by Esan et al. [85]. They proposed a rectangular spiral microchannel for separating particles of the same size as bacteria, e.g., *Escherichia coli* and *Staphylococcus aureus*, from heterogeneous debris extracted from ground meat and meat swabs (Figure 6e). When testing the device with polystyrene beads to mimic the average sizes of bacteria and debris (1.84, 6.04 and 10.6 μm) at a flow rate of 400 μL/min, they observed that 1.84 μm particles (a_p_/D_h_ ~ 0.03), which are comparable in size to bacteria, focused near the outer wall of the spiral channel; the 6.04 and 10.6 μm particles (a_p_/D_h_ ~ 0.15 and a_p_/D_h_ ~ 0.09) focused, instead, near the inner wall of the channel. Using the same device under similar experimental conditions but flowing bacterial and ground meats debris suspensions, they determined that the average percentage of debris collected was 49.4% at the inner outlet and 43.4% at the outer outlet, respectively. This result was likely due to the non-homogeneous nature of the debris containing particles smaller than 5 μm.

### 4.2. Trapezoidal Cross-Sectional Devices

Many optimizations of channel cross-section and other structural features have been made to enhance cell separation through inertial focusing. By increasing the height of the channel’s rectangular cross-section, Dean vortices become asymmetric, causing the vortex cores to shift towards the channel depth. 

**Figure 7:**
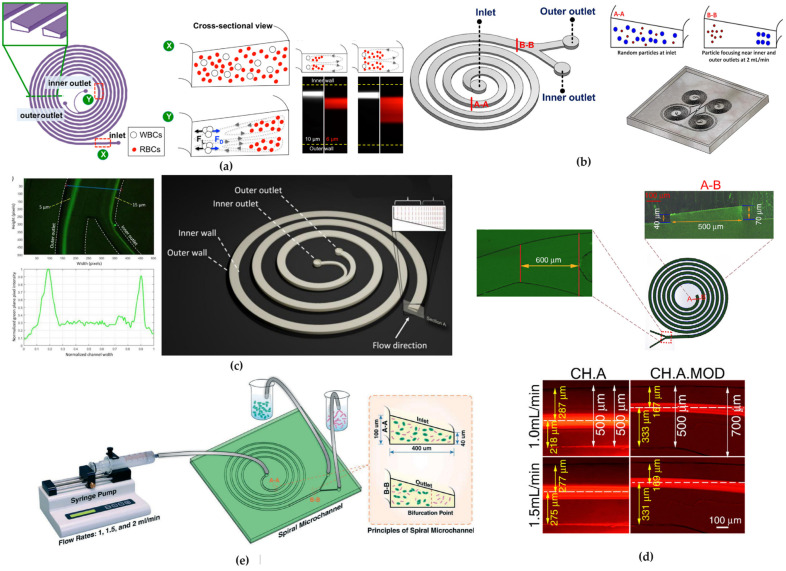
(**a**) Representation of a trapezoidal cross-section spiral microchannel illustrating the principle of particle focusing and trapping within the Dean vortices. Fluorescent images indicating the inertial focusing of 10 μm (white) and 6 μm (red) beads (reproduced from [57] under http://creativecommons.org/licenses/by/4.0/, accessed on 1 July 2024). (**b**) 3D drawing of a multiplex device of four spirals connected (reprinted with permission from [8]). (**c**) Inverter fluorescent microscope image of 5 and 15 μm particle separation in the trapezoidal device (reproduced from [49] under http://creativecommons.org/licenses/by/4.0/, accessed on 1 July 2024). (**d**) Schematic illustration of the experimental setup for inertial focusing of *S. pastorianus* and *L. brevis* along the inner and outer wall of the channel (reproduced from [1] under http://creativecommons.org/licenses/by/4.0/, accessed on 1 May 2024). (**e**) Cross-sectional image of the spiral microchannel with the wider outlet to improve the separation output (reprinted with permission from [80].

Building on this effect, in [57], a novel spiral channel with a trapezoidal cross-section with two asymmetric outlets was proposed for the first time (Figure 7a) to separate blood samples containing a high hematocrit content. This is often problematic due to cell–cell interactions affecting their focusing. The proposed approach involved increasing the spacing between equilibrium positions by fabricating a channel with higher depth towards the outer wall. It was also observed that, in addition to the criterion a/D_h_ > 0.07 [34], the height of the inner wall of the channel, was a critical parameter for determining the focus of the streamlines. Specifically, satisfying the criterion H_OUTER_/H_INNER_ > 1.5 was found to be essential. Polystyrene beads of 6, 10, and 15.5 µm size were separated at a flow rate of 0.8 mL/min. The device also demonstrated the ability to separate polymorphonuclear leukocytes (PMNs) and mononuclear leukocytes (MNLs) from diluted human blood with an efficiency greater than 80% at the same flow rate. Indeed, at a 0.8 mL/min flow rate, PMNs and MNLs formed a focused stream at a distance of approximately 75 μm away from the inner channel wall, while RBCs migrated to a broader stream near the outer channel wall due to their smaller cell size. The device was also used as a secondary step of differential centrifugation to further remove RBCs residuals from the isolated WBCs. 

Following Wu et al. [57], one of the earliest studies investigating the sorting mechanism of trapezoidal spiral microchannel was conducted by Guan et al. [53]. They compared three 8-loop single-inlet–two-outlet spiral channels: one with a trapezoidal cross-section and the other two with a rectangular cross-section. They evaluated particle separation of four different diameters (5.78 μm, 9.77 μm, 15.5 μm, and 26.25 μm) over flow rates ranging from 0.5 to 7.5 mL/min. They noticed that, beyond a threshold flow rate, depending on the radius of the spiral curvature and the slanted angle between the inner and the outer walls, there was an improved separation resolution of particles in the trapezoidal cross-section channel compared to the rectangular ones. They achieved separation of 15.5 µm from 18.68 µm beads with more than 92% efficiency. 

Warkiani’s group subsequently proposed two trapezoidal devices. First, they designed an 8-loop microchannel with a trapezoidal cross-section for sorting CTCs (breast adenocarcinoma, bladder and lung cancer cells) from RBCs in a blood sample, achieving an 85% efficiency with a blood flow rate of 1.7 mL/min [8]. Following this study, they proposed two 8-loop trapezoidal spiral microchannels with different dimensions to demonstrate the application of inertial spiral microfluidic system for the sorting of different types of cells, such as Chinese hamster ovary cell and *Saccharomyces cerevisiae* yeast cells (~3–5 μm). For CHO cells, they achieved separation of larger CHOs (~18 μm) from smaller CHOs (~11 μm) with an efficiency of 92% at a flow rate of 6 mL/min using a single-spiral device with cross-section dimensions of 600 μm in width and 80–130 μm in height. For yeast cells, a filtration efficiency of 90% at a flow rate of 2 mL/min was obtained using a trapezoidal microchannel with a width of 450 μm and heights of 30–70 μm. They also designed a multiplexed device combining multiple PDMS layers with spiral microchannels for continuous size-based separation of large sample volumes (Figure 7b) [54].

With the possibility of applying label-free spiral devices for separating a variety of cells having been proved, a trapezoidal cross-section microchannel was designed by Poon et al. [4] to isolate osteoprogenitor mesenchymal stromal cells (MSCs) from a heterogeneous culture bone marrow (BM) MSC population. Osteoprogenitor MSCs are interesting as candidates for tissue regenerative therapies, due to their self-renewing stem cells capabilities [99]. Size-based sorting enabled separating the osteoprogenitor subpopulation (~20 μm) from other MSC subpopulations (~15 μm) at a flow rate of 3.0 mL/min, though separation efficiency was not calculated in this study. 

Syed et al. [58] constructed an 8-loop trapezoidal spiral channel to separate and purify *Tetraselmis suecica* culture (a lipid-rich microalgae) from *Phaeodactylum tricornutum* (an invasive diatom). They first tested the device using 6 μm and 10 μm fluorescent polystyrene microbeads to simulate the behavior of microalgae cells. A separation efficiency ranging from 90 to 92% was demonstrated for both sizes of polystyrene microbeads at a flow rate of 1 mL/min. They observed that increasing the flow rate led to a widening of the particle focusing band, causing particles to exit through both outlets due to the action of $F_{Dean}$ force. When utilizing the microalgae solution, they achieved a separation efficiency of 90% for *P. tricornutum* and of 91% for *T. suecica* at a flow rate of 1 mL/min, with a 100% viability of both separated species. They also tested the trapezoidal microchannels to evaluate the dependence of concentration on separation efficiency, obtaining no significant improvement. In fact, higher concentrations led to possible re-contamination of the purified samples.

As in [4], Yin’s group [100] identified the “chondrogenic competent” subpopulation of human mesenchymal stem cells (MSCs) through size-dependent serial sorting using a trapezoidal spiral microchannel with asymmetric outlets. The target stem cells in culture had an average size between 17 and 21 μm. The pre-sorted population of MSCs was sequentially pumped through the device for four rounds of sorting. The flow rate was initially set at 3.5 mL/min to remove larger cell populations (>21 μm), and then decreased to 1.5 mL/min to remove smaller cell populations (<17 μm). This high-throughput sorting procedure enabled successfully isolating the medium-sized subpopulation (17–21 μm) from the heterogeneous total population, allowing for the expansion and proliferation of this specific subpopulation.

The study conducted by Al-Halhouli et al. demonstrated the effectiveness of trapezoidal spiral microchannel devices for inertial sorting [49]. They fabricated an 8-loop spiral microchannel fabricated using femtosecond laser ablation on glass (Figure 7c). The device was tested with 5, 10 and 15 μm fluorescent polystyrene microbeads. Through mathematical calculations, they predicted that at flow rates between 1 and 5 mL/min, for the 5 μm particles, $F_{Dean}$ was higher than $F_{L}$, while for the 10 and 15 μm particles, $F_{L}\;$ was higher than $F_{Dean}$. As confirmed by experimental results, the 5 μm particles focused at the core of the Dean vortices near the outer wall, while the larger particles focused near the inner wall, achieving high purity in particle separation. In a subsequent study, the same group [86] tested a second trapezoidal device with 2, 5, 10 μm fluorescent polystyrene microbeads. Experimental results demonstrated good separation of 2 from 5 μm particles at a flow rate of 0.6 mL/min and of 5 from 10 μm particles at a flow rate of 1 mL/min. They also found that below 0.3 mL/min, the focusing position was not stable for all particles. 

Promising results in particle sorting using trapezoidal cross-section channels were also achieved by Condina’s researchers’ group. They designed a trapezoidal spiral microchannel with asymmetric outlets (Figure 7d) for the differentiation of various cell types, including yeasts (*Saccharomyces pastorianus* and *Saccharomyces cerevisiae*) from beer spoilage microorganisms (*Lactobacillus brevis* and *Pediococcus damnosus*). *S. pastorianus* and *S. cerevisiae* have an elliptical shape, with average diameters of ~5 μm and ~4 μm, while *L. brevis* and *P. damnosus* have average diameters of ~2.8 μm and ~0.75 μm, respectively. In this study, larger particles (*S. pastorianus*) were focused on the inner wall, while smaller particles (*L. brevis*) were dispersed throughout the microchannel. To provide additional space for cells to focus, the inner wall was designed to be larger than the outer wall. The device was optimized using cultures in phosphate-buffered saline and lager beer: the focusing efficiency at the inner outlet for *S. pastorianus* was above 90% at flow rates of 1–2 mL/min, while for *L. brevis*, the efficiency was increased from 40% to 90% by repeating the separation process of the sample three times at 1.5 mL/min [1]. 

Further studies have explored how to modify the channel geometry to improve particle focusing and separation. For this reason, Mihandoust et al. [80] proposed two 6-loop microchannels with a trapezoidal cross-section, introducing a widening section at one of the loop’s channel outlet (Figure 7e). Testing the device to separate 4, 6 and 10 μm fluorescent microbeads, they observed that the centerline moved towards the inner wall, disrupting the balance between inertial lift and drag forces and pushing smaller particles towards the outer side of the channel. To compare the inertial focusing of both devices, they defined a sharpness factor *s* ($s=1-\frac{b-a}{W}$, where *b* is the width of particle focusing bands, *a* is the particle diameter, and *W* is microchannel width). The modified spiral microchannels led to a sorting efficiency of 98% for 6 μm particles at 1.5 mL/min, showing sharper focusing bands. 

In [101], inertial focusing in the spiral microchannel was employed for auto-perfusion in small-scale culture of suspension cells, aiming to replace conventional membrane filters that are subjected to clogging and fouling problems. They demonstrated the bioreactor system’s performance for culturing Chinese hamster ovary (CHO) cells (~17.7 μm). The inertial focusing mechanism was first tested with 15.45 μm polystyrene microspheres: at a flow rate of 1.5 mL/min, all the microspheres were sorted to the inner outlet, confirming focusing near the inner channel wall. The system enabled cell retention by focusing the cells to the inner outlet, reintroducing them into the cell culture vessel and removing the cell-free waste medium through the outer outlet.

In [72], Adel et al. conducted a study on the fabrication of a trapezoidal spiral microchannel using CO_2_ laser ablation for separating white blood cells (WBCs) from whole blood. For experimental validation, they tested the device’s separation efficiency at different blood dilutions to reduce the cell-to-cell interaction. They noted that increasing the hematocrit concentration (RBCs) lowered the sorting mechanism due to higher cell-to-cell interactions. With 1% hematocrits diluted blood sample, they achieved a 90.1% separation efficiency with an optimal flow rate of 800 μL/min and a WBCs recovery rate of 84%. 

Recently, in [102], Lu et al. proposed two-loop spiral microchannels for a trapezoidal cross-section for cell separation of A549 CTCs (~25 µm) from RBCs and WBCs (~6 and 15 µm, respectively) in blood samples. An initial numerical simulation showed that the larger A549 did not undergo Dean migration and focused on the inner wall of the channel, while the smaller RBCs and WBCs were subjected to Dean migration and focused to the outer wall of the channel. Numerical results allowed for experimental testing with 6 µm, 15 µm, and 25 µm polystyrene particles mimicking blood cells. A separation efficiency of 96.4% was obtained at an optimal flow rate of 1400 µL/min. Further tests were performed to explore the effect of particle concentration on sorting: separation of large particles increases with an increasing dilution due to stronger interactions at high concentrations. Finally, they validated the device with CTCs (A549) cells, obtaining a separation efficiency of 96.4%.

### 4.3. Triangular Cross-Sectional Devices

**Figure 8:**
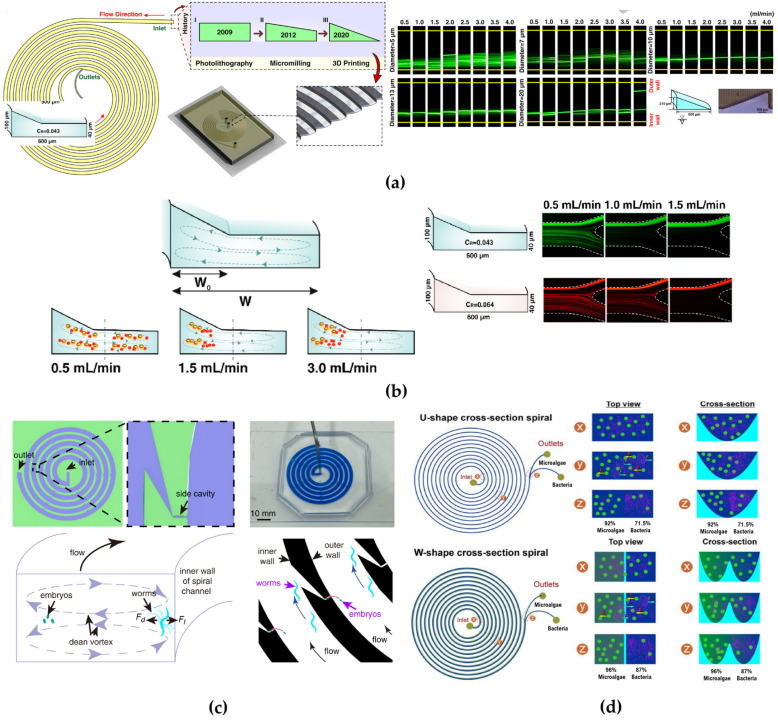
(**a**) Illustration of a right-angled triangular cross-section and images of 10 µm particles focused on a tight band at the outlet of the channel; double-band focusing appears at high flow rates (i.e., 4 mL/min) (readapted from [87] under http://creativecommons.org/licenses/by/4.0/, accessed on 10 May 2024). (**b**) Schematic of complex cross-section and lateral position for a differential displacement of particles at different flow rates; fluorescent images of the outlet showing the distribution of 4 µm (green) and 6 µm (red) beads at different flow rates (reproduced from [55] under http://creativecommons.org/licenses/by/4.0/, accessed on 1 July 2024). (**c**) Microfluidic device for sorting adult worms and embryos in the channel wall cavities (readapted from [84] under http://creativecommons.org/licenses/by/4.0/, accessed on 5 July 2024). (**d**) U-shaped and W-shaped cross-section channels for separation of microalgae and bacteria cells, with microalgae cells occupying the inner outlet and bacteria cells occupying the outer outlet (readapted from [2] under http://creativecommons.org/licenses/by/4.0/, accessed on 10 May 2024).

Recently, Bazaz et al. [87] fabricated an innovative spiral microchannel with a right-angled triangular cross-section and tested it with particles of 5, 7, 10, 13 and 20 µm in diameter (Figure 8a). Experimental results showed that particles larger than 10 µm focused in a single tight band at a flow rate of 4 mL/min, while for 20 µm particles, a double-band focusing appeared at the same flow rate. This study actually represents the only investigation on the focusing behavior of particles in triangular cross-sectional in spiral devices, likely due to the fabrication challenges of such a shape using the common techniques presented, in order to achieve channels with precise and sharp edges. The influence of triangular cross-sectional shapes on inertial focusing has been previously investigated in straight channels in the studies by [103,104]. 

### 4.4. Hybrid/Hybrid Cross-Sectional Devices

With the purpose of better understanding how the particles equilibrium positions and their focusing is affected by changing the microchannel cross-section, hybrid cross-section shapes, such as a combination of rectangular and trapezoidal geometry, have been investigated. In 2017, Ghadami et al. [59] proposed two geometries for a 4-loop spiral microchannel: (a) a rectangular cross-section and (b) a stair-like cross-section. Their study included both numerical simulation and experimental device characterization. Contrary to conventional rectangular spiral microchannels, where vortices form latitudinally, the stair-like design engineered vortices to be placed longitudinally next to each other. A correlation was found between the equilibrium position of vortices and the size-dependent flow rate threshold at which vortices stabilized themselves. The stair-like geometry allowed increasing the distance between separated particles, thus enabling high-throughput separation of 7.3 μm from 20 μm particles using a flow rate of ~2300 μL/min. 

In 2019, Raoufi’s group [75] designed a complex cross-section channel composed of two trapezoidal sections, which led to the formation of two bands, each located in one trapezoidal part. They suggested that the novel geometry could enable increasing the channel cross-sectional area for high-throughput applications. They tested the device for the separation and purification of mesenchymal stem cells (hMSCs) from microcarriers (with a dimension of ~180 µm) exiting from a perfusion bioreactor, reaching a 98% separation efficiency at a flow rate of 10 mL/min.

Mihandoust’s group [55] proposed a novel spiral microchannel with a complex cross-section consisting of a combination of trapezoidal and rectangular shapes, with the trapezoid section located in the inner region to isolate particles (4 and 6 μm) from the carrier fluid (Figure 8b). The purpose of this design was to create a wider cross-section channel to overcome the limitations of small channel cross-sections, which suffer from high fluidic resistance. To achieve cell sorting thanks to inertial forces, microchannels have to adhere to the $a/D_{h}>0.07$ criterion, which implies a very small cross-sectional area. To better understand the particle behavior in complex cross-sections, they defined a Complex Focusing Number (CFN) defined as $CFN=\cot\alpha \frac{w_{R}}{w_{T}}$ (where α is the angle between the sloping side and the horizontal line, $w_{R}$ is the width of the trapezoid section and $w_{T}$ is the width of the rectangular section). The higher the CFN number, the higher the threshold flow rate required for particles to exit their equilibrium positions. Numerical simulations showed that the complex cross-section led to narrower focusing bands due to increased fluid velocity in the trapezoidal region. With this design, a separation efficiency of approximately 98% was achieved.

Similar to the work in [55], Saha et al. [105] designed a complex cross-section channel with both rectangular and trapezoidal parts to improve isolation efficiency for the sorting of RBCs and WBCs from whole blood. Using computational fluid dynamics (CFD), they confirmed the potential of this hybrid cross-section of enhancing the sorting.

Another challenging work was recently published by Pan et al. [84]. They designed a rectangular cross-section spiral inertial microchannel for studying *Caenorhabditis elegans embryos* at different developmental stages (larvae/adult warms). The channel included side cavities, allowing particles close to the outer wall of the spiral channel to be trapped due to secondary Dean vortices, while particles close to the inner wall bypassed the side cavity (Figure 8c). This design allowed live imaging of single embryos by incorporating features for effective sorting and trapping. Considering the average hydraulic radii of *C. elegans* embryos and larvae, from ~24 to 78 μm, respectively, the channel dimensions were chosen to satisfy the a_p_/D_h_ > 0.07 criterion. They achieved separation of embryos from adult worms from a mixture of *C. elegans* with an efficiency of 85% at a flow rate of 1 mL/min.

Another novel cross-section geometry was designed by [2]. The group fabricated two spiral devices with U-shaped and W-shaped cross-section geometries to demonstrate the separation of microalgae (*Desmodesmus* sp. ~ 15 µm) from bacterial contaminants (*Escherichia coli* ~ 1 µm) during cultivation (Figure 8d). Experimental tests were conducted in the presence and absence of glycine. For the U-device, they achieved a separation efficiency of 92% for microalgae and 72% for bacteria with glycine, while 91% for microalgae and 63% for bacteria without glycine. For the W-device, they achieved a separation efficiency of 96% for microalgae and 87% for bacteria with glycine, and 96% for microalgae and 66% for bacteria separation efficiencies without glycine. Glycine was used as bacteria chemoattractant, providing extra energy for bacterial migration towards the target outlet. Additionally, the W-shaped cross-section, thanks to the barrier created at the center of the microchannels during laser ablation, prevented recirculation and mixing of microalgae and bacteria cells improving separation efficiency.

### 4.5. Combined Techniques Devices

Attempts to improve the performance of spiral microchannels have been made by combining inertial sorting technique to other techniques (i.e., passive or active).

**Figure 9:**
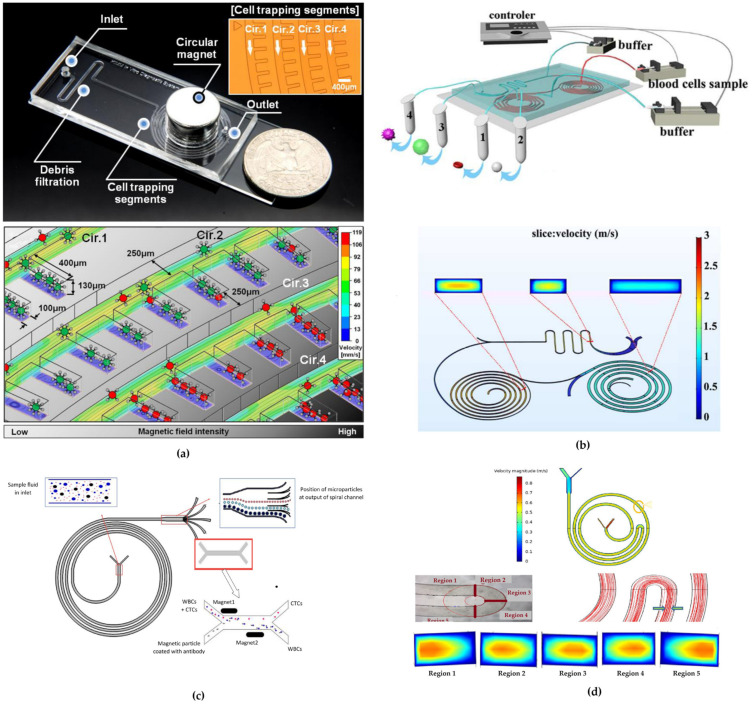
(**a**) Optical image of the spiral-shaped microfluidic channel with magnetic actuator and microscopic image of two types of breast cancer cells in trapping segments (reprinted with permission from [6]). (**b**) Schematic and simulation at the optimal flow rate of the cascade spiral and serpentine microfluidic channel (readapted with permission from [79]). (**c**) Illustration of the inertial and magnetic spiral sorter for separation of cancer cells from labelled WBCs (reproduced from [81] under http://creativecommons.org/licenses/by/4.0/, accessed on 10 July 2024). (**d**) U-shaped turn double-spiral microchannel geometric scheme and displacement of maximum velocity after and before the U-shaped turn (readapted from [82] under http://creativecommons.org/licenses/by/4.0/. accessed on 10 July 2024).

In 2017, Kwak’s group [6] developed a rectangular spiral microfluidic channel with trapping lateral chambers and a central magnet (Figure 9a). Their aim was to capture two different human breast cancer cell lines (MCF-7 ~ 15–17 μm [106] and MDA-MB-231 ~ 12 µm [107]) from blood cells through specific conjunction of Epithelial Cell Adhesion Molecule (EpCAM) to magnetic nanoparticles. As previously demonstrated by the same group [108], MCF-7 cells are non-metastatic while MDA-MB-231 cells are metastatic, with positive and negative expression, respectively. This allowed for differential conjugation differently with magnetic nanoparticles. The application of the magnetic field gradient enabled selective positioning of heterogenic CTCs. They achieved a separation efficiency of 97.2% for MCF-7 cells and 85.1% for MDA-MB-231 cells, respectively, with a flow rate of 150 μL/min.

An interesting study was conducted by Abdulla et al. [79], which aimed to achieve simultaneous separation of two differently sized CTCs (lung cancer cells and breast cancer cells) from blood cells (RBCs and WBCs). They proposed a multiplex cascaded microfluidic chip consisting of two 5-loop spiral channels of different dimension and a zigzag channel (Figure 9b). Deterministic lateral displacement (DLD) was used to combine inertial focusing in spiral channels, which are generally unable to separate particles or cells sized 20 µm and 25 µm from one another. The zigzag geometry enabled using the same flow rate across spiral sections, which is typically not compatible with conventional DLD channels.

One of the two spiral channels was connected both with the zigzag part and the other spiral part, which, respectively, ended with two outlets. Testing the device with polystyrene particles (5, 8, 15 and 24 μm) to mimic CTCs and BCs, they found that 5 and 8 μm particles were separated in the spiral channel at a flow rate of 2.2 mL/min, while 15 and 24 μm particles were separated in the zigzag part at a flow rate of 1.2 mL/min with high efficiency (≤97%). The device was also tested with RBCs-lysed diluted human blood samples, performing the separation of WBCs and lung cancer cells from breast cancer cells. Tabatabaei et al. [81] sought to enhance the performance of inertial microfluidic devices and increase the purity of isolated cells by serially integrating a spiral microchannel with a straight microchannel equipped with magnetic actuators. Magnetic separation is an active technique where the target cells are labelled with magnetic beads by antibodies and then separated by applying a magnetic field gradient, while unlabeled cells follow the flow direction. For this reason, this technique can offer more precise control over cells allowing organization of labelled cells [12]. Their 4-loop rectangular cross-section spiral microchannel was designed to separate RBCs and WBCs with smaller size from CTCs, followed by a second separation step using magnetic beads for active separation to improve CTC isolation (Figure 9c). Evaluating the device with 5 μm and 15.3 μm monodisperse particles, they achieved separation efficiencies of 80% and 86%, respectively, at a flow rate of 1200 µL/min.

Another study by Kumar et al. [109] demonstrated inertial focusing of 10 µm and 15 µm particles using three different spiral microchannel devices with rectangular cross-sections. The first device consisted in a 10-loop spiral microchannel with a rectangular cross-section used to investigate particle behavior with increasing channel length and number of loops and consequently, the separation distance. The device showed stable particles focusing at the outer wall of the 10th loop of the microchannel at flow rates of 400 µL/min. The second device, consisting of a single two-loop spiral microchannel, achieved particle separation efficiencies of 98% for 10 µm particles and 97% for 15 µm particles at a low flow rate of 0.1 mL/min. The third device integrated two spiral microchannels connected by a U-shaped turn to handle higher throughput: the first spiral was used for pre-focusing the particles, while the second spiral enhanced migration and separation. This configuration achieved separation efficiencies of 89% for 10 µm particles and 99% for 15 µm particles at a flow rate of 1 mL/min. The device design provided high throughput while minimizing sample volume, which is crucial for clinical applications requiring large sample processing.

The study also examined the effect of varying the viscoelastic properties of the fluid on lateral particle focusing by adding different concentrations of Polyethylene Oxide (PEO) as an elasticity enhancer. The authors observed that higher PEO concentrations increased fluid viscosity and enhanced both inertial and elastic lift forces, improving particle migration and focusing, while low concentrations resulted in weaker focusing.

Further attempts to combine multiple channel geometries were made by Omrani’s group [82], who proposed a novel pattern with U-shaped turn combining spiral trapezoidal cross-section and serpentine patterns to improve the focusing of different sizes cells. Changes in curvature ratio, by introducing a U-shaped turn, can influence the direction and magnitude of Dean flow, as well as the maximum velocity location, which depends on the Reynolds number, the curvature ratio, and the cross-section (Figure 9d). At the U-shaped turn, the maximum velocity shifted from the outer wall into the inner wall. As a consequence, when particles moved from the channel segment before the U-shaped turn to the segment after, larger particles migrated from the outer wall to the inner wall, while smaller particle moved close to the outer wall, increasing the distance between their respective equilibrium positions.

The device was tested with a mixture of monodisperse (5 and 15.6 μm) and polydisperse (2–20 μm) microparticles, obtaining a 94% focusing efficiency at a flow rate of 1.7 mL/min. Then, the spiral microchannel was further validated to separate breast cancer cells (BCCs, ~11–15–18–21 μm) from WBCs (~6–16 μm) (Figure 9d). Introducing the U-shaped turn, numerical simulations showed a larger distance between the larger from the smaller particles after the U-shaped turn. Experimental results demonstrated that WBCs and CTCs focusing was possible at a flow rate of 1.7 mL/min, with isolation efficiency of approximately 92% and 93%, respectively. 

In 2023, Gucluer’s group [77] published a study demonstrating the possibility of fabricating a low-cost microfluidic device using rapid laser ablation (with a CO_2_ laser) on PMMA. The device was designed by combining a serpentine section and a spiral microfluidic channel with a rectangular cross-section to achieve the separation of *Saccharomyces cerevisiae* yeast cells and bacteria. To simulate the average cell dimensions, they validated the device with polystyrene particles of 1 µm and 5 µm in diameter. The serpentine section was used to focus the particles into a narrow stream before they entered the spiral microchannels, without the use of sheath flow. Experimental tests with polystyrene particles demonstrated separation efficiencies of 93% and 89% for the 1 µm and 5 µm microparticles, respectively, at a flow rate of 800 µL/min. The authors observed that at flow rates above 1000 µL/min, there was a decrease in separation efficiency, with failure occurring at 3000 µL/min. In further validation tests with *Saccharomyces cerevisiae* yeast cells and 1 µm beads, used to mimic bacteria, they achieved separation efficiencies of 91% and 85% for yeast cells and 1 µm microbeads, respectively.

**Table 3:** Characteristics of spiral microfluidics studies.

Loop	Channel Dimensions	Particles’ Size	Flowrate	Separation Efficiency (%)	Ref.
Rectangular cross-section					
>10	W = 100 µm H = 1250 µm	Polystyrene beads: 1, 8, 10 µm	2 mL/min	Not specified	[66]
>5					
5	W = 100 µm H = 50 µm	Polystyrene beads = 7.32 and 1.9 µm	10 μL/min	100%	[78]
5	W = 500 µm H = 130 µm	Polystyrene beads: 10, 15, 20 µm	3 mL/min	90%	[45]
	W = 500 µm H = 120 µm	SH-SY5Y neuroblastoma cells and C6 glioma cells		80%	
4	W = 500 µm H = 150 µm	RBCs ~ 7 µm WBCs ~ 10–20 µm	1.8 mL/min	95%	[35]
	W =250 µm H =75 µm	Polystyrene beads: 7.32, 10, 15 and 20 µm	1–3 mL/min	All in the first outlets/no separation	
4	W = 150 µm H = 50 µm	Sperm cells ~ 9 µm RBCs ~ 9 µm	0.52 µL/min	81–99%	[98]
3 × 2	W = 500 µm H = 170 µm	Polystyrene beads: 6, 10 and 15 µm	100 µL/min	90%	[50]
		CTCs > 15 µm WBCs ~ 7–15 µm	3 mL/h (0.1 mL/min)		
7	W = from 200 to 600 H = 100 µm	Polystyrene beads: 7, 10 and 15 µm	1.6 mL/min	72%	[89]
		Human dendritic cells ~ 10–15 µm RBCs ~ 7 µm WBC ~ 7–15 µm			
1	W = 500 µm H = 200 µm	RBCs ~ 7 µm CTCs (HeLa and MCF-7) ~ 16–24 µm	~113–139 mL/h	100%	[94]
2 × 5	W = 300 µm H = 100 µm	Polystyrene beads: 6, 10, 20 and 40 μm *algal dinoflagellate* species ~ 20–60 µm	2000 µL/min	>94% (loss < 6%)	[83]
2	W = 200 µm H = 70 µm	Polystyrene beads: 1.84, 6.04 and 10.6 μm	400 µL/min	50%	[85]
		Bacteria *(Escherichia coli*/*Staphylococcus aureus)* ~ 1 µm ground meat debris			
Trapezoidal cross-section					
8	W = 500 µm H_in_ = 70 µm H_out_ = 100 µm	Polystyrene beads: 6,10, 15.5 µm in water	0.8 mL/min	>80%	[57]
		Polymorphonuclear leukocytes (PMNs), mononuclear leukocytes (MNLs) and haematocrits			
8	W = 600 µm H_in_ = 80 µm H_ou_ = 130 µm	Polystyrene beads: 5.8, 9.8, 15.5, 26.25 µm	0.5–7.5 mL/min	92%	[53]
8	W = 600 µm H_in_ = 80 µm H_ou_ = 130 µm	RBCs ~ 7 µm Different CTCs ~ 15–20 µm	1700 µL/min	Not specified	[8]
8	W = 600 µm H_in_ = 80 µm H_ou_ = 130 µm	Polystyrene beads: 10 µm and 15 µm	6 mL/min	92%	[54]
		Mammalian cells: Chinese hamster ovary cells ~ 10–20 µm			
	W = 450 µm H_in_ = 30 µm H_ou_ = 70 µm	Polystyrene beads: 4 µm	2 mL/min	90%	
		Yeast cells: *Saccharomyces cerevisiae* ~ 3–5 µm			
8	W = 600 µm H_in_ = 80 µm H_ou_ = 130 µm	Mesenchymal stem cells (hMSCs) ~ 11–25 µm	3 mL/min	Not specified	[4]
8	W = 600 µm H_in_ = 80 µm H_out_ = 130 µm	Polystyrene beads: 6 µm and 10 µm	1 mL/min	80–91%	[58]
		Microalgae: *Tetraselmis suecica* ~ 10.7 µm; *Phaeodactylum tricornutum* ~ 25.7 and 3.5 µm			
8	W = 580 µm H_in_ = 85 µm H_out_ = 133 µm	Mesenchymal stem cells (hMSCs) ~ 11–25 µm	1.5 mL/min	Not specified	[100]
8	W = 600 µm H_in_ = 50 µm H_out_ = 90 µm	Polystyrene beads: 5, 10 and 15 µm	1–5 mL/min	Not specified	[49]
8	W = 200 µm H_in_ = 40 µm H_out_ = 90 µm	Polystyrene beads: 2, 5 and 10 µm	0.6–1 mL/min	Not specified	[86]
4	W = 400 µm H_in_ = 40 µm H_out_ = 100 µm	Beer Spoilage Bacteria ~ 2–5 µm	1.5 mL/min	90%/ > 50%	[1]
6	W = 500 µm H_in_ = 40 µm H_out_ = 70 µm	Polystyrene beads: 4, 6 and 10 µm	1.5 mL/min	98%	[80]
6	W = 600 µm H_in_ = 80 µm H_out_ = 130 µm	Polystyrene beads: 15.45 µm	1.5 mL/min	Not specified	[101]
		Chinese hamster ovary cells ~ 17.7 µm			
8	W = 600 µm H_in_ = 110 µm H_out_ = 70 µm	RBCs ~ 7 µm WBCs ~ 10–15 µm	800 µL/min	90%	[72]
2	W = 500 µm H_out_ = 150 µm H_in_ = 75 µm	Polystyrene beads: 6 µm, 15 µm, and 25 µm	1400 µL/min	96.4%	[102]
		RBCs ~ 6 µm; WBCs ~ 15 µm; A549 CTCs ~ 25 µm			
Triangular cross-section					
5	W= 600 µm H_max_ = 210 µm H_min_= 0 µm	Polystyrene beads: 5, 7, 10, 13 and 20 µm	4 mL/min	Not specified	[87]
Hybrid/complex cross-section					
Stair-like					
4	W_1_ = 500 µm H_1_ = 110 µm W_2_ = 100 µm H_2_ = 70 µm	7.32 and 20 μm Human umbilical vein endothelial cells (HUVEC) and fibroblast cells	2300 μL/min	Not specified	[59]
Rectangular + double trapezoidal					
5	D_h_ = 250 µm	Microcarriers ~ 180 µm Mesenchymal stem cells (hMSCs) ~ 15–30 µm	10 mL/min	98%	[75]
Rectangular + trapezoidal					
4.5	W = 400 µm H_in_ = 40 µm H_out_ = 100 µm	Polystyrene beads: 4, 6 and 10 µm	1.5 mL/min	Not specified	[55]
	W = 500 µm H_in_ = 40 µm H_out_ = 100 µm			Not specified	
	W = 600 µm H_in_ = 40 µm H_out_ = 100 µm			97–98%	
Rectangular + cavities					
5	W = 1600 µm H = 50 µm	*C. elegans embryos* ~ 24 µm *adult worms* ~ 26, 32, 40, 61 and 78 μm	1 mL/min	85%	[84]
U shaped and W shaped					
10	W = 227 µm H_max_ = 210 µm H_min_ = 175 µm	Microalgaes *(Desmodesmus* sp.) ~ 15 µm bacteria (*Escherichia coli*) ~ 1 µm	0.7 mL/min	92–72%	[2]
	W = 220 µm H_max_ = 210 µm H_min_ = 162 µm			96–66%	
Combined techniques devices					
Rectangular spiral + cavities + magnetic actuator					
3.5	W = 250 µm H = 130 µm	*Breast cancer cell lines* (MCF-7 ~ 15–17 μm and MDA-MB-231 ~ 12 µm) BCs	150 μL/min	~97–85%	[6]
Rectangular spiral + DLD					
2 × 5	W = 200 µm H = 80 µm	Polystyrene beads: 5, 8, 15, and 24 µm	1.2 mL/min– 2.2 mL/min	≤97%	[79]
		RBC: 7.34 µm—WBC: ~12 µm *Lung cancer cells* (A549): ~10–15 µm *Breast cancer cells* (MCF-7): ~15–25 µm			
Rectangular spiral + magnetic actuator					
4	W = 500 µm H = 130 µm	Monodisperse beads: 5 and 15.6 μm Polydisperse beads: from 2 to 20 μm	1200 μL/min	86–80%	[81]
Rectangular spiral + U-shaped turn					
4	W = 500 µm H = 180 µm	Polydisperse beads: 10 µm; monodispersed beads: 5 µm and 15 µm	1.7 mL/min	93%	[82]
Rectangular spiral + U-shaped turn					
10	W = 500 µm H = 50 µm	Polystyrene beads: 10 and 15 µm	>400 μL/min	/	[109]
2	W = 500 µm H = 50–200 µm		0.1 mL/min	98–97%	
2 + U	W = 500 µm H = 100 µm		1 mL/min	89–99%	
Rectangular spiral + DLD					
3	W=/H = 100 µm	Polystyrene beads: 1 and 5 µm	800 μL/min	89–93%	[77]
		*Saccharomyces cerevisiae* yeast cells		91–85%	

## 5. Conclusions and Perspectives

This review presents various studies focused on understanding how different types of cells can be separated in spiral microfluidic channels. The state-of-the-art materials and fabrication techniques for this type of chip are also reviewed. Articles reporting studies on chips with different channel cross-sections are discussed, demonstrating that this passive technique can be a valuable and versatile method for the separation and filtration of cells of various sizes and for different applications.

Although more research is needed to fully understand the physical mechanisms—such as how different-sized and -shaped particles reach equilibrium positions depending on the channel’s cross-section and flow speed—this technique appears promising. Spiral devices using inertial sorting can potentially meet the ASSURED criteria proposed by the World Health Organization (WHO). Key points include

1. **Affordable and Delivered**: The use of inexpensive, easy-to-process, recyclable, durable, non-toxic, and biocompatible materials (such as PDMS and PMMA) is crucial for the affordability and practicality of these devices. Future advancements may focus on incorporating eco-friendly materials that enhance sustainability and performance. Researchers are exploring new polymers and composite materials that could further improve the durability, biocompatibility, and environmental impact of spiral microfluidic devices.
  Rapid manufacturing techniques contribute to the affordability and scalability of these devices. While traditional soft lithography is widely used, it has limitations including time-consuming fabrication steps and lower precision for complex geometries. Future developments may see increased use of additive manufacturing (3D printing) for precise fabrication of intricate designs. Despite limitations in material availability, advancements in 3D printing technologies could expand the range of usable materials. Additionally, ultrashort laser ablation, though less common, offers high precision and speed and is compatible with various materials, including PMMA. The focus will likely be on refining these techniques for large-scale production and reducing material waste.
2. **Equipment-free, User-friendly and Robust**: Spiral inertial microfluidic devices are considered equipment free because they are microfluidic structures on small-scale single- or multi-layer chips that do not require external mechanical or electrical forces. This contributes to their user-friendliness and robustness. Future improvements could focus on optimizing channel dimensions and cross-sectional geometries to enhance sorting efficiency and throughput. The passive sorting mechanism relies on intrinsic fluid dynamics, which eliminates the need for additional mechanical or electronic equipment. This simplicity, combined with the ability to operate under high flow pressure conditions, makes these devices highly robust and suitable for a variety of applications.
3. **Sensitive and Specific**: Future research should aim to better match channel and cross-section dimensions with specific cell sizes to enhance sensitivity and specificity in sorting techniques. By fine-tuning these parameters, inertial spiral microchannels could achieve higher throughput and efficiency. Ongoing studies will likely explore how to optimize these devices for different types of cells and particles, potentially leading to more precise and effective sorting capabilities.

As technology advances, spiral microfluidic devices may find broader applications in medical diagnostics, environmental monitoring, and industrial processes. Future developments could include integration with other analytical techniques and sensors, enabling multifunctional devices capable of performing complex assays and analyses. The potential for on-chip integration with real-time imaging and data analysis systems could further enhance the utility and applicability of these devices.

## Data Availability

Data will be made available on request.
